# Personalized External Aortic Root Support (PEARS) in the Treatment of Marfan Syndrome and Bicuspid Aortic Valve Aneurysms: First Case Series in the American Continent

**DOI:** 10.21470/1678-9741-2024-0370

**Published:** 2025-09-11

**Authors:** Renato A. K. Kalil, Felipe Borsu de Salles, Cristiano Blaya Martins, Rafael Ceron, Lucas Krieger Martins, Eduardo Menegat, Conal Austin, Claudia Ciceri Cesa, Tal Golesworthy

**Affiliations:** 1 Instituto de Cardiologia do Rio Grande do Sul, Fundação Universitária de Cardiologia, Porto Alegre, Rio Grande do Sul, Brazil; 2 Hospital Moinhos de Vento, Porto Alegre, Rio Grande do Sul, Brazil; 3 Hospital Divina da Rede de Saúde Divina Providência, Porto Alegre, Rio Grande do Sul, Brazil; 4 St Thomas' Hospital, London, United Kingdom; 5 Instituto Federal Sul-Rio-Grandense, Pelotas, Rio Grande do Sul, Brazil; 6 Exstent Ltd., Tewkesbury, United Kingdom

**Keywords:** Marfan Syndrome, Aortic Root Aneurysm, Cardiopulmonary Bypass, Pyrus, Atrial Fibrillation, Coronary Vessels, Anticoagulants, Pericarditis.

## Abstract

**Introduction:**

Conventional surgical approaches for aortic root aneurysms, including valved grafts and valve-sparing techniques, present inherent limitations such as the requirement for anticoagulation and the potential for late reoperation. Personalized External Aortic Root Support (PEARS), utilizing the ExoVasc® implant, represents a novel approach that aims to overcome these limitations.

**Methods:**

This report presents the initial clinical experience with the ExoVasc® PEARS implant in the Americas, encompassing 10 patients (six males, age range 30 - 52 years, mean age 37.8 years) diagnosed with aortic root aneurysms. Indications for PEARS included Marfan syndrome (eight patients, including one reoperation), bicuspid aortic valve (two patients, including one with anomalous coronary artery), and associated valvular dysfunction. Cardiopulmonary bypass was utilized in four cases.

**Results:**

No major adverse postoperative events were observed. Postoperative recovery was generally uneventful, with minor complications, including pericarditis and atrial fibrillation, successfully managed with medical therapy. Aortic dimensions remained stable at 30-day and one-year follow-ups.

**Conclusion:**

This initial experience demonstrates the feasibility, safety, and efficacy of the PEARS technique for the treatment of aortic root aneurysms. Potential advantages over traditional approaches include the possibility for off-pump procedures, reduced risk of aortic valve dysfunction, shorter hospital stays, and elimination of the need for long-term anticoagulation therapy. Further investigation is warranted to evaluate the long-term durability and clinical outcomes of this innovative approach.

## INTRODUCTION

**Table:** 

Abbreviations, Acronyms & Symbols	
AR	= Aortic regurgitation
BAV	= Bicuspid aortic valve
CPB	= Cardiopulmonary bypass
ICU	= Intensive care unit
PEARS	= Personalized External Aortic Root Support

Ascending aortic aneurysms involving the sinuses of Valsalva and the aortic valve, such as those seen in Marfan syndrome, and other connective tissue disorders (*e.g.*, bicuspid aortic valve disease, heritable genetic diseases) are progressive and carry risks of valve insufficiency, dissection, rupture, and death. These aneurysms frequently occur in young patients, and surgical intervention is often indicated based on the degree of aortic dilation, even before significant regurgitation develops^[1,2]^.

Surgical management of these aneurysms typically involves either the Bentall-DeBono procedure, which replaces the affected aorta and valve with a valved conduit, or valve-sparing techniques like the Tirone David and Magdi Yacoub procedures. These latter techniques preserve the patient's native valve by incorporating it into a Dacron® graft^[3]^.

The ExoVasc® Personalized External Aortic Root Support (PEARS) implant is a patient-specific, custom-made graft that reinforces the aorta externally, eliminating the need for resection^[4]^. Compared to the Bentall-DeBono procedure, PEARS preserves the native aortic root and valve, avoiding the lifelong anticoagulation required with mechanical valve replacement. Furthermore, unlike the David and Yacoub techniques, PEARS avoids direct valve manipulation, potentially reducing the risk of future valve-related complications and reoperations. The PEARS graft can be customized to precisely match the patient's aortic dimensions or undergo a diametral reduction, typically around 95% of the original diameter. This reduction strategy serves the dual purpose of decreasing the aortic diameter while simultaneously bringing the aortic valve commissures closer. This optimized positioning facilitates leaflet coaptation, thereby reducing/eradicating any dilation-induced aortic valve regurgitation that may be present.

The technique is expanding as a viable treatment option. The initial clinical cohort was published showing design, manufacturing, and implantation of PEARS, with an increasing cumulative number of patients operated and low morbidity and short length of stay^[5]^. This technique is being proposed and recognized by numerous publications^[6,7]^ and has approval for clinical use in countries in Europe, Asia, and Oceania. The PEARS Project registry completed 20 years in 2024 and had 1,224 patients enrolled by February 2025^[8]^, of which 1020 for Aortic PEARS and 204 for Ross-PEARS. On the American continent there was no series of cases carried out until 2022.

This report presents the first results of PEARS cases implemented in Brazil under feasibility projects. We describe the surgical procedures and clinical outcomes, including up to two years of follow-up data.

## METHODS

This research was approved as a pilot study for feasibility and safety by the Comissão Nacional de Ética em Pesquisa (or CONEP) under numbers CAAE 54699421.9.0000.5330 at Hospital Moinhos de Vento and CAAE 28882620.2.0000.5333 at Instituto de Cardiologia.

A series of 10 patients who were operated on between March 2022 and November 2023 is reported (Table 1). The age range was 30 to 52 years with a mean of 37.8 years. There were seven males and three females.

**Table 1:** Patients’ clinical data and demographics.

Number of patients	10
Age, mean (min. - max.)	38 years (30 - 53)
Sex	7 males/3 females
Condition (Marfan or other)	80% Marfan/20% BAV
Aneurysm diameter (mm), mean (min. - max.)	50 (45 - 62)
Aortic regurgitation (before)	70% none/30% mild
Previous cardiac surgery	10% (1 ascending aortic graft interposition)
CBP used (yes/no)	60% no/40% yes
Concomitant procedures	2 patients
	(1 mitral repair and 1 anomalous circumflex coronary artery dissection and osteoplasty)
Operation time (min.) (min. - max.)	260 (140 - 600)
CPB time (min.) (min. - max.)	119 (64 - 235)
Death	0
Stroke	0
Myocardium infarction	0
Reoperation	0
Dialysis	0
Aortic valve preservation	100%
Aortic regurgitation (after)	80% none/20% trace
Length of hospital stay (days), mean (min. - max.)	7 (5 - 11)
Intraoperative adverse events	90% no/10% yes
Adverse events in ICU	90% no/10% yes
Technically successful procedure	100% yes

BAV=bicuspid aortic valve; CPB=cardiopulmonary bypass; ICU=intensive care unit

Marfan syndrome was the most common underlying condition, present in eight patients, one of whom had undergone a previous operation. Two other patients had ascending aortic aneurysms associated with bicuspid aortic valves. In one of these cases, the left circumflex coronary artery originated anomalously from the right sinus of Valsalva. Two patients presented with mild aortic insufficiency, and one had severe mitral insufficiency. The mean aortic diameter was 50 mm (range 45 - 62 mm). The implanted grafts were sized at 95% of the aortic diameter in nine patients, and at 80% of the aortic diameter in one case, *i.e.*, a diametral reduction of 5% in nine cases and 20% in one case.

### Surgical Technique

**Fig. 1:**
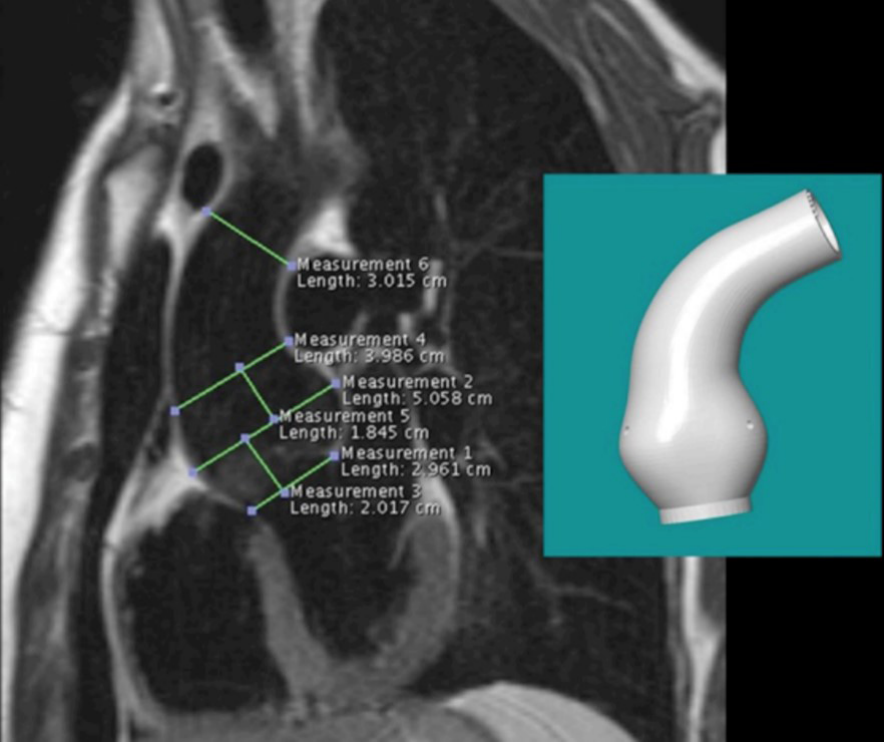
Image of the patient’s aorta illustrating some of the key measurements made, together with an inset photograph of the resulting three-dimensional model used as a mold in the manufacture of the Personalized External Aortic Root Support (or PEARS) mesh.

The operations were conducted as previously described by Pepper et al.^[4]^. The chest was opened through a median sternotomy. The aorta was completely dissected from the aorto-ventricular junction to the origin of the brachiocephalic artery, under controlled systemic hypotension. Cardiopulmonary bypass (CPB) was available but only used if necessary. The ExoVasc® PEARS graft was brought to the operating table on the mold, a model of the patient’s own aorta (Figure 1). Accommodations for the coronary arteries were cut into the ExoVasc® graft which was then removed from its mold by opening the longitudinal seam sited over the non-coronary sinus and the support placed around the aorta. Note that the material covers the aortic sinuses of Valsalva, proximal to the coronary arteries, down to the aorto-ventricular junction (Video 1). It is engineered to have high hoop strength preventing annular dilatation.

**Video 1:**
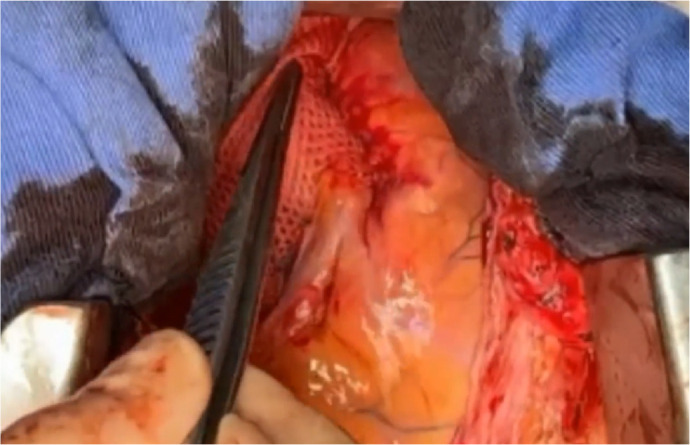
Final transoperative result of ExoVasc PEARS implant in a 29-year-old Marfan’s female patient, showing complete covering of the aortic root and ascending aorta, from the aortic-ventricular junction up to the origin of the brachiocephalic arterial trunk and the origins of both coronary arteries arising through the implant.

*Link: https://youtu.be/7hW9mQArkxQ*

CPB was required in four patients: one for mitral valve repair, one to address an anomalous origin of the left circumflex coronary artery, one for a reoperative procedure due to a previous ascending aortic graft, and one due to aortic wall fragility and adhesions following a recent episode of chest pain. The remaining six procedures were performed off-pump. Off-pump procedures were completed within three to 4.5 hours, while surgeries utilizing CPB were generally longer due to the treatment of associated conditions.

## RESULTS

No immediate postoperative mortality or major adverse events, including stroke, myocardial infarction, renal failure, or the requirement for reoperation, were observed in this series. In six patients diagnosed with Marfan syndrome presenting with typical aneurysms and no prior surgical interventions, the procedure was successfully completed without CPB use. The patient with concomitant mitral regurgitation necessitated use of CPB following aortic graft placement for mitral valve repair. In the patient with a history of a prior ascending aortic graft, CPB was employed to facilitate dissection. The existing graft was preserved and incorporated into the PEARS graft, which extended proximally to the aortic-ventricular junction.

A patient with Marfan syndrome presented with a 62 mm aortic diameter, exhibiting a 7 mm dilation over the past 18 months. This patient experienced an episode of acute chest pain one month prior to the PEARS surgery. The posterior aortic wall was found to be adherent to the pulmonary artery, necessitating the use of CPB for dissection. Reduction aortoplasty was performed, followed by graft implantation. This complex procedure lasted approximately 10 hours (235 minutes on CPB) but was completed without complications, and the patient was discharged home 10 days later.

A complex surgery was performed on a 30-year-old patient with a 45 mm aortic diameter, bicuspid aortic valve, and an anomalous left circumflex coronary artery originating from the right coronary sinus. The patient experienced significant symptoms including chest pain and pallor with moderate exertion. During surgery, the coronary artery was carefully dissected from the aortic wall, the ostium was repaired, and the PEARS graft was successfully implanted.

The postoperative course was uneventful, with only two minor complications observed: pericarditis and atrial fibrillation. Both conditions were successfully resolved with medical management. Aortic valve regurgitation was present in four patients preoperatively (trace in two and mild in two). Transesophageal echocardiography demonstrated a significant reduction in regurgitation, with none detected in eight patients and trace regurgitation in two. This favorable outcome persisted at 30 days and during long-term follow-up (Table 2).

**Table 2:** Aortic valve insufficiency before and after PEARS.

	Pre	Post
	*n*	*n*
Absent	6	8
Trace	2	2
Mild	2	0

PEARS=Personalized External Aortic Root Support

**Fig. 2:**
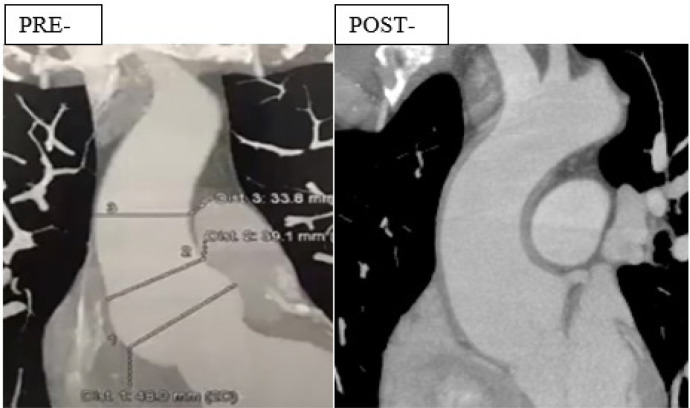
Preoperative and one-year postoperative computed tomography angiography of the first case in this series, a 41-year-old male patient with FBN1 mutation confirmed Marfan’s syndrome, aortic root aneurysm with 48 × 48 mm diameter, and mild aortic insufficiency that was completely corrected by the implanted graft. Immediately and one year postoperatively, there was no aortic regurgitation at echo examination and the aorta was stable at 44 × 44 mm maximum diameter at the sinuses. Observe the shadow of thickening of the aortic wall determined by the implanted graft, from the aortic ventricular junction, below the sinuses of Valsalva, up to the origin of the brachiocephalic trunk and the near normal postoperative aortic shape.

Hospital stay ranged from four to 10 days, with an average of 6.8 days. Computed tomography angiography at 30 days and one year postoperatively demonstrated stable aortic dimensions (Figure 2).

## DISCUSSION

This report presents the initial experience of PEARS implantation in Brazil, a pioneering initiative within the Americas. Novel surgical techniques require rigorous evaluation to demonstrate safety and reproducibility. PEARS enables ascending aortic aneurysm repair in the majority of cases without the need for CPB, resulting in low in-hospital mortality and morbidity^[8]^. By scaling graft diameter to reduce aortic diameter, it may be possible to enhance aortic valve function by bringing the valve leaflets closer together, thereby improving their coaptation in those cases where aortic regurgitation (AR) is dilation induced.

This study demonstrated excellent early postoperative outcomes with no in-hospital major adverse events in a small cohort of patients. All patients had short intensive care unit stays with no stroke, myocardial infarction, need for dialysis, reoperation, prolonged mechanical ventilation, or major bleeding. Postoperative complications were limited to two minor events: pericarditis and atrial fibrillation. Both conditions were successfully managed with medical therapy. These results align with global PEARS experience, which reports a remarkably low surgical mortality rate (*i.e.*, 0.1%)^[8]^. This low mortality rate underscores the safety profile of PEARS, particularly compared to other surgical series with higher mortality rates, varying from 0.7 to 9.7%^[9-13]^. Some previous Brazilian publications had higher mortality rates, ranging between 2.9% and 7.5%^[14,15]^. The favorable outcomes observed in this study support the potential for expanded utilization of the PEARS technique.

Long-term follow-up data^[8]^ includes one patient at 20 years, 44 patients at 10 years, and 292 patients at five years. Only one death, attributed to ischemic heart failure, was observed during the PEARS follow-up period. In contrast, other surgical series exhibit a wide range of long-term mortality rates, with five-year mortality ranging from 6.3%^[16]^ to 15.1%^[10]^ and 10-year mortality ranging from 10.5%^[16]^ to 17%^[9]^. Long-term mortality in these series is primarily attributed to neoplasia, cardiac failure, and the need for reoperation. Importantly, the PEARS cohort demonstrated superior long-term outcomes with no aortic dilatation, dissection, or aortic-related deaths during follow-up.

This initial Brazilian series demonstrated improved aortic valve function following PEARS implantation, consistent with findings from previous studies. Notably, AR was reduced, with only two of 10 patients exhibiting trace regurgitation postoperatively. This finding underscores the significant advantage of the PEARS technique: the preservation of native aortic valve function, thereby eliminating the need for valve replacement and the associated risks of anticoagulation therapy or long-term prosthetic degeneration.

Freedom from reoperation is crucial for patients with long life expectancies, particularly those with conditions like Marfan syndrome. Historical data from Bentall and David procedures demonstrates a 10-year freedom from reoperation rate as high as 89.5% and 87.8%, respectively^[16]^, with higher rates of serious bleeding observed after Bentall procedures. The global PEARS experience demonstrates superior freedom from reoperation, with only two reoperations required (after six and nine years). These reoperations were necessitated by the development of significant AR in patients with incomplete PEARS mesh coverage due to intraoperative factors, constituting a deviation from the established protocol. Importantly, no reoperations were required for aortic-related causes.

PEARS implantation has primarily been established in Europe, Asia, and Oceania. This Brazilian series represents the first reported clinical application of the PEARS technique in the Americas. The introduction and clinical utilization of PEARS in the United States of America and Canada remain subject to regulatory approval.

### Limitations

This is a small clinical series of cases operated by the same surgical team and represents an initial local experience. The good results are supported by previous experience of other centers and by proctoring by Mr. Conal Austin in every procedure. It is assumed that further experience would maintain the expected outcomes, as training and expertise are developed.

## CONCLUSION

The treatment of aortic root aneurysms with PEARS presents a feasible, safe, and effective alternative to conventional surgical techniques. Compared to traditional procedures such as the Bentall-DeBono, Yacoub, and David techniques, PEARS offers advantages, including the ability to perform the procedure without the need for extracorporeal circulation, the avoidance of direct valve manipulation, and the potential for reducing valve insufficiency. Furthermore, PEARS is associated with shorter operative and hospitalization times, elimination of the need for long-term anticoagulation therapy and potential for improved long-term outcomes with a reduced risk of late aortic events.
